# Treatment of Fracture-Related Infection after Pelvic Fracture

**DOI:** 10.3390/jcm12196221

**Published:** 2023-09-27

**Authors:** Viola Freigang, Nike Walter, Markus Rupp, Moritz Riedl, Volker Alt, Florian Baumann

**Affiliations:** 1 Department of Trauma Surgery, University Medical Centre Regensburg, Franz-Josef-Strauss-Allee 11, 93053 Regensburg, Germany; 2 Faculty of Interdisciplinary Studies, Landshut University of Applied Sciences, Am Lurzenhof 1, 84036 Landshut, Germany

**Keywords:** pelvic fracture, fracture-related infection

## Abstract

Background: The management of pelvic fractures is a significant challenge. Surgical site infection can result in the need for revision surgery, cause functional impairment, and lead to a prolonged length of stay and increased treatment costs. Although reports on fracture-related infection (FRI) after pelvic fracture fixation are sparsely reported in the literature, it is a serious complication. This study analysed patients with FRIs after pelvic fracture regarding patient characteristics, treatment strategies, and an evaluation of risk factors for FRI. Methods: In this retrospective single-centre study, FRI was diagnosed based on clinical symptoms of infection and a positive culture of a bacterial infection. Depending on the severity and acuteness of the infection, osseous stabilization was restored either via implant retention (stable implant, no osteolysis), exchange (loose implant or bony defect), or external fixation (recurrence of infection after prior implant retaining revision). Healing of infection was defined as no sign of recurring infection upon clinical, radiological, and laboratory examination in the last follow-up visit. Results: The FRI rate in our patient population was 7.5% (24/316). In 8/24 patients, the FRI occurred within the first three weeks after initial surgery (early) and 16/24 presented with a late onset of symptoms of FRI. A strategy of debridement, antibiotics, and implant retention (DAIR) was successful in 9/24 patients with FRI after pelvic fracture. A total of 10 patients required an exchange of osteo-synthetic implants, whereof three were exchanged to an external fixator. In five patients, we removed the implant because the fracture had already consolidated at the time of revision for infection. A total of 17/24 patients had a poly-microbial infection after a pelvic fracture and 3/24 patients died from post-traumatic multi-organ failure within the first 6 months after trauma. There were no cases of persistent infection within the remaining 21 patients. Conclusions: Although poly-microbial infection is common in FRI after pelvic fracture, the recurrence rate of infection is relatively low. A complex pelvic trauma with significant soft tissue injury is a risk factor for recurrent infection and multiple revisions. A strategy of DAIR can be successful in patients with a stable implant. In cases with recurrent infection or an unstable fracture site, the exchange of implants should be considered.

## 1. Introduction

A fracture of the pelvic ring is a serious and potentially life-threatening injury. The management of pelvic fractures requires a specialized facility and a multidisciplinary team-based approach [1]. Pelvic ring fractures occur in up to 20% of severely injured patients and have a mortality rate of up to 10% [2,3,4]. Pelvic ring fractures are often a result of high-energy trauma and lead to mechanical instability [5]. Successful treatment of unstable and displaced fractures first consists of damage control and temporary pelvic stabilization with pelvic binders, followed by either external or internal fixation, pelvic packing, and embolization [6]. For definitive operative stabilization of the pelvic ring, extensive surgical approaches may be necessary to achieve good reduction and stable fixation [7]. Numerous perioperative and postoperative complications can occur after emergency treatment [8]. The occurrence of a fracture-related infection (FRI) is a serious complication that occurs in 2.1–9.2% of pelvic fractures [9]. However, fracture-related infection after pelvic fracture treatment remains generally an under-reported issue in the literature. FRI can lead to unexpected revision surgery and severe socioeconomic and personal impacts on the patient [10]. The patient’s quality of life decreases as social participation and mobility declines [11]. Functional impairment and a prolonged length of hospital stay may be consequences of a revision surgery due to an FRI. In addition to the patient-related consequences, FRIs may increase short-term and long-term healthcare costs [12]. Studies have shown that the cost for patients who sustain an FRI is up to six times higher than in patients with the same fracture pattern without subsequent infection, with the prolonged length of stay at the hospital mainly contributing to the cost. The recurrence rate of an FRI is up to 9%, causing unexpected re-admission rates [13].

Establishing the ideal treatment strategy is challenging since diagnosis and treatment strategies depend highly on location and individual patient characteristics. In 2018, a consensus meeting established a clear definition of FRI to standardize clinical studies and improve the overall quality of FRI research [14]. The consensus group defined two levels of certainty of diagnostic features. The criteria are confirmatory or suggestive of the presence of an FRI. The detection of representative criteria should lead to further investigation and, ideally, confirmatory criteria. Affirmative criteria are sinus tract, wound breakdown, and pus; phenotypically indistinguishable pathogens identified through culture from two or more separate deep tissue specimens; and microorganisms in deep tissue on histopathological analysis. Suggestive criteria are clinical signs, radiological/nuclear imaging signs, joint effusion, persistent wound drainage, elevated serum markers, and pathogens identified from just one deep tissue sample [14].

This study aims to evaluate the diagnosis, treatment, and short-term outcomes of patients with FRIs after pelvic fracture treatment. We want to identify patient characteristics, assess the risk for FRI in fractures, and describe a multidisciplinary treatment strategy for FRIs in pelvic fractures.

## 2. Methods

This retrospective single-centre study evaluated all patients with an FRI after pelvic fracture between July 2012 and June 2021. FRI was defined as published by the FRI consensus group. [15] Based on our clinical information system, we identified 24 patients with an FRI after pelvic fracture treatment. The inclusion criteria were:

- Age over 18;
- Pelvic fracture treatment with open reduction and internal fixation;
- FRI diagnosed according to clinical symptoms of infection AND a positive culture of a bacterial infection;
- A complete dataset regarding preoperative and postoperative computed tomography.

The exclusion criteria were:

- Isolated external fixator treatment (pin track infection);
- Missing data or lack of consent to participate in the study.

We characterized the patients by demographics, fracture pattern, and concomitant injuries. Fractures were classified according to the AO Classification or the Rommens Classification according to the mechanism of injury.

### 2.1. Initial Surgical Management and After-Care

The initial management of the pelvic fracture was performed according to the AO (Arbeitsgemeinschaft für Osteosynthesefragen) principles. The timing of the surgery was dependent on the patient’s condition and concomitant injuries. There was an indication for open reduction and fixation depending on the degree of instability of the pelvic ring (e.g., complete fracture of the posterior pelvic ring or ligamentous injury). All patients received a postoperative computed tomography (CT) of the pelvis for an evaluation of the quality of reduction and implant position. We administered 2 g Cefazolin as a peri-operative i.v. antibiotic prophylactic. Postoperative after-care included partial weight-bearing for six weeks on the side where the posterior pelvic ring was disrupted and radiographic follow-up after 6 weeks; 3, 6, and 12 months; and until the consolidation of the fracture. In cases of clinical symptoms of infection, laboratory examination (including CRP levels and leukocyte cell count), as well as ultrasound-guided fluid aspiration (including microbiology and cell count), and CT of the pelvis, were part of the protocol for diagnosing FRI in pelvic fractures.

### 2.2. Surgical Management of Fracture-Related Infection

**Figure 1:**
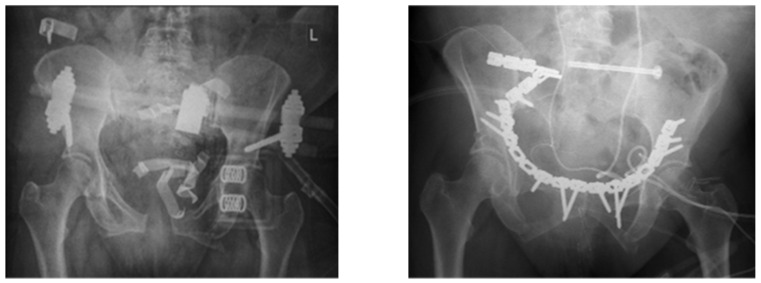
A 57-year-old female patient with a C-type pelvic fracture. Infection of the dorsal plates 3 months after the trauma. Successful treatment with DAIR.

For revision surgery for FRI after pelvic fracture, we used the former approach to perform radical debridement around the implant and the former fracture site (Figure 1). After scar resection and reopening the surgical site, we took at least five deep tissue samples from the soft tissue and the affected bone for histopathology and cultures. After radical debridement of inflammatory and necrotic tissue, irrigation with saline solution was executed. If fracture healing was incomplete by the time of occurrence of FRI, we implemented a procedure including debridement, antibiotics, and implant retention (DAIR). Depending on the severity and acuteness of the infection, osseous stabilization was restored either via implant retention (stable implant, no osteolyses), exchange (loose implant or bony defect), or external fixation (recurrence of infection after prior implant retaining revision).

The postoperative protocol consisted of suction drainage for 48 h. The standard antibiotic therapy included intravenous therapy for two weeks and oral antibiotics for another 4–12 weeks. Empiric antibiotic therapy consisted of Vancomycin, which was replaced with targeted treatment as soon as intraoperative culture results were obtained.

Laboratory as well as clinical symptoms of infection were monitored post-operatively. In case of recurrence of FRI, further revisions with the debridement and exchange of implants were scheduled. The number of debridements performed depended on the severity of the infection and acuteness of the symptoms (acute = symptoms less than 3 weeks; chronic = duration of symptoms more than 3 weeks). The regression of clinical symptoms and normal laboratory findings were conditions for healing infection. All patients received anticoagulation prophylaxis according to individual patient needs. Laboratory as well as clinical symptoms of infection were monitored closely until discharge. The follow-up visits after infection treatment were scheduled for six weeks, 12 weeks, six months, and one year and consisted of X-rays, clinical examination, and blood testing.

The healing of infection was defined as no sign of recurring infection upon clinical, radiological, and laboratory examination in the last follow-up visit.

### 2.3. Statistical Analysis

Statistical analysis was performed using the software package SPSS (Version 28, SPSS Inc., Chicago, IL, USA). For the comparison of mean values, we used the independent *t*-test. For ordinal data, we used the Chi-square test. We used the Wilcoxon rank test for non-parametric comparison testing. Unless otherwise stated, descriptive data are given as the mean ± standard deviation. The level of significance was at *p* < 0.05 for all tests.

The Ethics Committee at the University of Regensburg approved the study in December of 2022 (Institutional Review Board Number 22-3175-104). All procedures performed in this study were in accordance with the Declaration of Helsinki (1964).

## 3. Results

### 3.1. Demographics and Clinical Characteristics

During the study period between June 2012 and July 2021, our institution treated 316 patients surgically for a pelvic fracture. Twenty-four patients (7.5%) developed an FRI. Table 1 shows the baseline characteristics of the patients. There were 9 women and 15 men in the study group, with a median age of 58.1 years (20.1–79.6 years). The median follow-up period was 6.2 years (IQR 5.24 years). ASA physical status was 2 (10× ASA 2, 12× ASA 3, and 2× ASA 4) in the study population. Most patients in the study group endured a high-velocity injury, with 5 patients suffering from a pelvic ring fracture AO Type B and 12 patients from an AO Type C injury. In seven cases, an osteoporotic fragility fracture of the pelvis occurred prior to the FRI. We classified four patients as fragility fracture type Rommens IIIa, two patients as type Rommens IIIb, and one patient as Rommens type IVa. (Table 1). A total of 22/24 patients had undergone open reduction and open fixation of the pelvic fracture. Two patients developed an FRI after non-operative treatment of a pelvic fracture. A total of 5/24 patients (all high-energy trauma) were haemo-dynamically unstable at admission and required initial pelvic packing and staged revision after 24–48 h. Additionally, 8/24 patients with an FRI had sustained a complex trauma of the pelvis with a significant soft tissue injury. Among those, there were two patients with a traumatic hemipelvectomy and one more case with a dissection of the iliac artery. There were three patients who sustained a traumatic rupture of the bladder, three patients for whom the prostate was involved, and two patients with an injury of the sigmoid colon.

### 3.2. Treatment Strategies and Risk Factors for Recurrent Infections

A total of 6/24 patients sustained recurrent infection with the need for a second revision for FRI. All of these patients had a complex pelvic trauma initially with soft tissue injury and the involvement of the bladder or bowels. Soft-tissue laceration was the only risk factor the patients with a recurrence of infection and multiple revisions had in common. These patients received inter-disciplinary (together with visceral surgery, vascular surgery, urology, or gynaecology) reconstruction and staged second-look surgeries.

In 8/24 patients, the FRI occurred within the first three weeks after initial surgery (early), and 16/24 presented with a late onset of symptoms of an FRI. A strategy of debridement, antibiotics, and implant retention (DAIR) was successful in 9/24 patients with an FRI after pelvic fracture. Three patients required an exchange of osteosynthetic implants, whereof three were exchanged to an external fixator. In five patients, we removed the implant because the fracture had already consolidated at the time of revision for infection.

### 3.3. Microbiology and Anti-Infective Therapy

Table 2 shows the results of the microbiology analysis of the 24 patients. A total of 17/24 patients had a poly-microbial infection after pelvic fracture. We had to isolate 6/18 patients for a multi-resistant bacteria (VRE/MRGN) infection. In three patient samples, we found an additional fungal infection. Piperacillin/Tazobactam, Vancomycin, Ciprofloxacin, Meropenem, and Linezolid were the most frequently used antibiotics. Additionally, 3/24 patients died from post-traumatic multi-organ failure within the first 6 months after trauma. There were no cases of persistent infection within the remaining 21 patients.

## 4. Discussion

Fracture-related infection is a serious complication in pelvic fracture management. Our study reports on short-term results after FRI indicate that the recurrence rate of infection is relatively low even though analysis of the microbiology results revealed poly-microbial infection in most cases. All patients with recurrent infection and multiple revisions had sustained a complex pelvic trauma with significant soft tissue injury initially. Regarding surgical management of the infection, only 7/24 patients required internal re-stabilization of the fracture site with the exchange of implants. A strategy of DAIR was successful in 9/24 patients; in all other cases, the fracture site was stable enough to remove the implants or otherwise treat with external fixation.

Even though consensus guidelines have benefited the research community immensely, investigating FRIs in specific regions for the general quest for optimal patient-centred quality of care is necessary. We report on 24 patients who sustained FRI after the open reduction and internal fixation of a traumatic pelvic ring fracture. When developing a treatment strategy, FRI-specific aspects, fracture patterns, soft tissue damage, and patient-related factors should be considered. The prevalence of FRI varies between 2–30% and increases with certain patient-related risk factors [16]. These include diabetes, old age, and immune deficiency, which are associated with anti-infective treatment failure [13,17,18,19,20]. Increasing age also increases the risk of fracture-related infection [13,21]. The elderly are shown to have a slower healing process in all phases of wound healing. Due to the decrease in proliferation, remodelling takes more time, and the resulting collagen is inferior [22]. However, we did not observe any association between recurrent infection and age or diabetes. The initial soft tissue injury was the only parameter the patients with recurrent infection had in common. Evaluation of the initial soft tissue injury and early adaptation of the surgical treatment strategy may be important clinical implications for FRIs in pelvic fracture management.

In contrast to peri-prosthetic joint infection (PJI), FRI treatment guidelines are limited, and due to the complexity of the cases, the application of standardized protocols is difficult. Therefore, a patient-related, multidisciplinary approach with surgical and non-surgical specialists is necessary to develop an individual treatment strategy. In addition to surgical considerations, the involvement of an antibiotic stewardship program is advisable. It improves the quality of care while enhancing patient safety and outcomes and contributes to a reduction in re-admission rates and healthcare costs [10,23]. This approach requires interdisciplinary cooperation. In our institution, we established a meeting of all parties involved in infection prevention and treatment to discuss all current cases of FRI and establish an individual treatment strategy. In the bigger picture, this also improves the prevention of FRI in general as it enhances susceptibility to the complexity of the problem, which requires a systematic approach, with attention to risk factors and improvement of the patient’s public health and immune defence.

When managing FRI in pelvic ring fractures, soft tissue damage, severe concomitant internal injuries, vascular compromise, perioperative illness, and multiple surgeries are among the challenges surgeons face [24]. The necessary internal fixation in pelvic ring fractures provides an ideal environment for infection [25]. The extent of soft tissue damage and vascular compromise determines microbial infiltration, which negatively influences bony healing. Perren’s concept of strain theory and fracture healing predicts poor neovascularization in unstable fractures. Osteolysis and soft tissue damage further contribute to the likelihood of bacterial growth, immune compromise, and further instability of the fracture [26,27]. Trauma itself, as well as surgical intervention, causes immune suppression. Concomitant injuries, embolization, retroperitoneal packing, closed internal de-gloving, urogenital and bowel tract injuries, prolonged operative time, soft tissue injury, open pelvic fractures, loss of blood, and admission to intensive care also contribute to the probability of infection. A main strength of this study is the strict inclusion criteria to include only culture-positive infection cases. In agreement with another study, we found a variety of bacteria in the cultures taken [9]. However, neither the type of bacteria nor the number of different bacterial strains had an impact on the recurrence of infection as long as the antibiotic treatment was targeted to the bacterial strain.

Since bacteria which form biofilm survive higher antibiotic dosages, adequate treatment consists of surgery and antibiotics. A mature biofilm forms over the course of a few weeks and has a significant influence on the efficiency of antibiotic therapy. Injury severity (ISS) and the extent of soft-tissue trauma, according to the Gustilo–Anderson classification, are measurable variables to identify patients at risk for FRI. Especially in those patients, infection prevention is of utmost importance and should include soft tissue coverage and systemic and local antibiotic therapy [28]. Open fracture wounds should be covered as soon as possible, preferably within three days [29]. Prophylactic first-generation cephalosporins have a protective effect concerning FRIs and, when facing severe tissue damage, can be combined with broad-spectrum Gram-negative antibiotics. The duration of prophylactic systemic antibiotic treatment is a topic of ongoing discussion. We usually administer only single-dose antibiotics before surgery in cases of closed fractures without tissue damage. In Gustilo–Anderson I/II cases, we opt for 24 h of antibiotic prophylaxis with a maximum of 3 days in cases of severe tissue damage [30].

Bony consolidation is significant for curing the infection since osteosynthesis cannot permanently be removed without challenging fracture healing. Whether to opt for implant retention or exchange depends on several factors. Firstly, extensive debridement is a must. It reduces the bacterial load, local and systemic inflammation, and therefore enhances the performance of antibiotics [31]. Two surgical treatment concepts are to be considered, but randomized controlled trials in the treatment of FRI are scarce. The first concept is debridement, antibiotics, and implant retention (DAIR). Studies have shown that the bone is not prone to osteomyelitis within the first two weeks of osteosynthesis, even in cases of colonized implant material [29]. The second concept includes debridement, antibiotics, and either implant exchange or removal depending on fracture healing. Stable fracture fixation is necessary to achieve recovery from infection. Even though infection is considerably more likely in the presence of osteosynthesis, experimental studies have shown that a fracture without fixation is even more susceptible to infection [32,33]. Facing the problem of choosing implant removal or retention, several factors should be considered, including the quality of soft tissue, fracture stability, the ability of thorough debridement, anatomic localization, perfusion, patient-specific factors, and the time between the onset of infection and osteosynthesis [30]. Various studies have shown the outstanding results of DAIR in the first three weeks, but the success declines drastically after six weeks as biofilm formation matures, and antibiotics are less likely to penetrate and eradicate the infection [34,35,36]. In our collective, we removed the osteosynthesis in 15 cases out of 24 patients. In 9/24 patients, a single DAIR procedure was successful for the treatment of an FRI of the pelvis. The overall excellent success rate of both DAIR and implant removal or exchange could be explained by the localization of the fracture and consecutive infection. Infection can be life-threatening, and functional limb loss or amputation leads to decreased functional status [10]. The pelvic region possesses two key aspects that enhance success rates in FRI: good vascularization and soft tissue coverage [37]. Both contribute to an environment that prevents further contamination, ensures antibiotic delivery, and enhances fracture healing [30]. However, the extent of vascularization and soft tissue may vary based on the soft tissue injury. The extent of the initial “trauma zone” may play an important role in FRI, as we observed recurrence of infection only in complex pelvic trauma patients.

A main limitation of this study is the small number of patients. However, the number of pelvic fractures is limited, even in large trauma centres, and the complication rate for deep infection is below 10%. We only included patients with a positive culture in a fluid or tissue sample of the fracture site. There may be more culture-negative infections; however, they may not be clinically relevant. However, this is the first study providing detailed information on demographics, surgical management, and microbiological findings in FRIs in pelvic fracture care. We advocate for further registry-based studies on infection after pelvic fracture to evaluate this severe complication in a larger number of patients to reduce bias.

Secondly, the retrospective study design results in limited data for evaluation. Fracture-related infections and consecutive treatment put a lot of stress on patients. The prolonged hospitalization and repeated operations influence the patient’s physical and mental health. Health-related quality of life (HRQoL) is a neglected parameter in patients with FRI. HRQoL may deteriorate rapidly when a complication like FRI occurs. Studies have shown that even years after successful surgical treatment of an FRI, patients experience significantly lower HRQoL [38]. Therefore, we advocate for prospective studies to evaluate HRQoL in patients with FRI after pelvic fracture management.

## 5. Conclusions

Short-term results after FRI indicate that the recurrence rate of infection is low even though poly-microbial infection is a common microbiological finding. All patients with recurrent infection and multiple revisions had sustained a complex pelvic trauma with significant soft tissue injury initially. A strategy of DAIR can be successful in patients with a stable implant. In cases with recurrent infection or an unstable fracture site, the exchange of implants should be considered. However, further research is necessary to confirm these results and evaluate patient-centred HRQoL outcomes after FRI following pelvic fracture. We recommend further registry-based studies on infection after pelvic fracture for a better understanding of this severe complication in a larger number of patients. 

## Figures and Tables

**Table 1:** Demographics and initial management (ASA = American Society of Anesthesiologists classification; Fix ex = fixateur externe; TIFI = trans-iliac internal fixateur).

n = 24	High-Energy Trauma (n = 17)		Low-Energy Trauma (n = 7)	Total
Age	51.8 (±20.7)		74.8 (±7.3)	58.1 (±20.9)
Gender	5 female/12 male		5 female/2 male	9 female/15 male
ASA Score	8× ASA 2 7× ASA 3 2× ASA 4		2× ASA 2 5× ASA 3	10× ASA 2 12× ASA 3 2× ASA 4
Fracture Classification	AO/OTA 5× B-type, 12× C-type		Rommens 4× IIIa, 2× IIIb, 1× IVa	
Stabilization Method	anterior	15× plate	1× plate	16× plate
		1× Fix ex	4× Fix ex	5× Fix ex
	posterior	7× screws	3× screws	10× screws
		2× plate	2× plate	4× plate
		3× TIFI	2× TIFI	5× TIFI
		2× lumbo-pelvic	1× lumbo-pelvic	3× lumbo-pelvic

**Table 2:** Microbiology results of fluid or tissue samples taken from the fracture site.

Microbiology Results	n =
*Staphylococcus epidermidis*	9
*Staphylococcus aureus*	7
*Staphylococcus haemolyticus*	2
*Staphylococcus lugdunensis*	2
*Staphylococcus caprae*	1
*Staphylococcus warneri*	1
*E. coli*	5
*E. faecium*	5
*Proteus mirabilis*	3
*Enterobacter cloacae*	2
*Klebsiella*	2
*Stenotrophomonas maltophilia*	1
*Pseudomonas aeroginosa*	1
*Propionibacterium species*	1
*Cutibacterium acnes*	1
*Bacillus megaterium*	1
*Corynebacterium amylonatum*	1
*Fusarium*	1
*Bacillus cereus*	1
*Picha norvegensis*	1
*Citerobacter freundii*	1
*Candida albicans*	2
*Aspergillus flavus*	1
Cultural combinations in 17 poly-microbial infection cases	
- *Citerobacter freundii* + *Picha norvegiensis* + *Bacteroides* sp. + *Aspergillus flavus*	
- *Staph haemolyticus* + *E. faecium* + *Candida albicans*	
- *Staph.* epi + *E. coli*	
- *Staph.* epi + *Proteus mirabilis*	
- *Staph.* epi + *Pseudomonas aerogenosa* + *Proteus mirabilis*	
- *Staph.* epi + *Staph haemolyticus* + *E.faecium*	
- *Stenotrophomonas maltophilia* + *Corynebacterium amylonatum* + *Bacillus cereus* + *Fusarium* spp. + *Candida albicans*	
- *Staph. aureus* + *Staph.* epi	
- *Staph. aureus* + *E. coli*	
- *Staph epi* + *Staph aureus* + *Klebsiella*	
- *Staph aureus* + *Enterobacter cloacae*	
- *Staph.* epi + *Staph caprae* + *Klebsiella* + *E. faecium*	
- *Staph.* epi + *E. cloacae*	
- *E. coli* + *E. faecium*	
- *Staph. aureus* + *Bacillus megaterium*	
- *Staph.* epi + *E. faecium* + *Proteus mirabilis*	
- *Staph aureus* + *Staph. warneri*	

## Data Availability

Data are available on reasonable request to the corresponding author.
